# Reproductive and Hormonal Factors in Relation to Lung Cancer Among Nepali Women

**DOI:** 10.3389/fonc.2019.00311

**Published:** 2019-05-07

**Authors:** Sanah N. Vohra, Amir Sapkota, Mei-Ling T. Lee, Chin B. Pun, Binay Thakur, Bhola Siwakoti, Paddy L. Wiesenfeld, Mia Hashibe, Cher M. Dallal

**Affiliations:** ^1^Department of Epidemiology, Gillings School of Global Public Health, University of North Carolina, Chapel Hill, NC, United States; ^2^Division of Applied Regulatory Toxicology, Office of Applied Research and Safety Assessment, Center for Food Safety and Applied Nutrition, U.S. Food and Drug Administration, Laurel, MD, United States; ^3^Department of Epidemiology and Biostatistics, University of Maryland School of Public Health, College Park, MD, United States; ^4^Maryland Institute for Applied Environmental Health, University of Maryland School of Public Health, College Park, MD, United States; ^5^B. P. Koirala Memorial Cancer Hospital, Bharatpur, Nepal; ^6^Division of Public Health, University of Utah School of Medicine, Salt Lake City, UT, United States

**Keywords:** lung cancer, Nepal, women, reproductive factors, hormonal factors

## Abstract

**Background:** Of the 1.8 million global incident lung cancer cases estimated in 2012, approximately 60% occurred in less developed regions. Prior studies suggest sex differences in lung cancer risk and a potential role for reproductive and hormonal factors in lung cancer among women. However, the majority of these studies were conducted in developed regions. No prior study has assessed these relationships among Nepali women.

**Methods:** Using data from a hospital-based case-control study conducted in B. P. Koirala Memorial Cancer Hospital (Nepal, 2009–2012), relationships between reproductive and hormonal factors and lung cancer were examined among women aged 23–85 years. Lung cancer cases (*n* = 268) were frequency-matched to controls (*n* = 226) based on age (±5 years), ethnicity and residential area. The main exposures in this analysis included menopausal status, age at menarche, age at menopause, menstrual duration, gravidity, and age at first live-birth. Odds ratios (ORs) and 95% confidence intervals (CIs) were estimated using multivariable logistic regression.

**Results:** Among postmenopausal women, those with a younger age at menopause (<45 years; 45–49 years) had an increased odds of lung cancer compared to those with an older (≥50 years) age at menopause [OR (95%CI): 2.14 (1.09, 4.17); OR (95% CI): 1.93 (1.07, 3.51)], after adjusting for age and cumulative active smoking years. No statistically significant associations were observed with the other reproductive and hormonal factors examined.

**Conclusion:** These results suggest that Nepali women with prolonged exposure to endogenous ovarian hormones, via later age at menopause, may have a lower odds of lung cancer.

## Introduction

Lung cancer is the most commonly diagnosed cancer and the primary cause of cancer mortality worldwide (1–4). Moreover, approximately 60% of the global lung cancer incidence and mortality occurs in less developed regions (LDRs) (3), which include countries such as Nepal. Although lung cancer is one of the three most common cancers among Nepali women (5), epidemiologic data on the risk factors of lung cancer in LDRs remains scarce (6, 7).

While smoking is a well-established risk factor for lung cancer, several studies have suggested that reproductive and hormonal factors may also play a role in lung cancer development due to observed sex differences (8–12). These sex differences include increased susceptibility to the carcinogenic effects of tobacco (8–10) and a higher proportion of non-smoking lung cancer diagnosed (11) among women, as compared to men. Additionally, the distribution of histological subtypes differ by sex with adenocarcinoma being more common among women vs. squamous cell carcinoma in men (13). The presence of female sex-hormone receptors (estrogen-β and progesterone) on lung cancer cells (14) along with the patterns noted above support hypotheses related to reproductive and hormonal factors and lung cancer.

Prior epidemiological studies which investigated the relationship between age at menarche (15–27), age at first birth (15, 16, 18–25, 27, 28), and oral contraceptive use (17, 19–22, 24, 27, 29) in relation to lung cancer risk have reported no statistically significant associations, while only one study to date has examined the relationship between gravidity and lung cancer and observed no statistically significant association (16). More recent cohort studies conducted in the United States have observed an inverse association between lung cancer risk and age at menopause (20–23), and longer reproductive periods (22, 24) lending support to these potential associations. Despite these limited prior studies, reproductive and hormonal factors have not been examined in relation to lung cancer among Nepali women, an understudied population with differential patterns of these exposures.

Given the increased lung cancer global burden in LDRs (1, 2) such as Nepal, the suggested higher susceptibility among women (8–12), and the above-mentioned biological rationale, relations between reproductive and hormonal factors and lung cancer among Nepali women were examined. We hypothesized that the odds of lung cancer among Nepali women may differ by factors such as age at menarche, age at menopause, menstrual duration, number of pregnancies or gravidity, and age at first live-birth.

## Methods

### Study Population

This study utilizes data from a hospital-based case-control study, conducted between November 2009 and December 2012, at the B. P. Koirala Memorial Cancer Hospital (BPKMCH) in the city of Bharatpur, Chitwan district (30, 31). The primary study is described in previous publications (30, 31).

Briefly, 606 lung cancer cases and 606 frequency-matched controls, based on age (±5 years), sex, ethnicity, and residential area (district), were recruited, including 268 female lung cancer cases and 226 female frequency-matched hospital-based controls. One lung cancer case was excluded due to a previous breast cancer diagnosis. Thus, the final analytic population for this study included 493 women (267 cases and 226 controls).

Lung cancer was defined according the International Classification of Diseases for Oncology, 2nd Edition (ICD-O2) codes C33 (trachea) and C34 (bronchus and lung). Eligible cases included patients diagnosed with primary lung cancer at the BPKMCH. Eligible controls included visitors (family and friends) of non-lung cancer patients, subjects being screened for cancer and subjects accompanying the person being screened, at the BPKMCH during the study period. Although the primary study aimed to frequency-match cases and controls based on sex, hospital visitors were less likely to be women, and thus, fewer female controls were recruited. Lung cancer patients younger than 18 years of age and not residing in Nepal for at least 5 years were excluded.

All eligible participants were interviewed in-person by a trained nurse, who collected detailed information including tobacco and alcohol consumption, reproductive and hormonal factors, medical conditions, family history of cancer, anthropometrics and other factors using a standardized questionnaire. A target interval of 1 day and a maximal interval of 3 months between diagnosis and recruitment were used for data attainment in order to minimize selection bias. Informed written consent was obtained from the study participants prior to enrollment. Institutional Review Board approvals were obtained from the University of Utah, University of Maryland and the Nepal Health Research Council.

### Exposure Assessment

Reproductive and hormonal exposures assessed include menstrual duration (continuous in years), menopausal status (premenopausal, postmenopausal), age at menarche (<14, 14, ≥15 years), age at menopause (<45, 45–49, ≥50 years), number of pregnancies (≤2, 3–5, >5), age at first live-birth (<18, 18–20, ≥21 years) and birth control use (ever/never). Among postmenopausal women, menstrual duration was defined as the difference between the participants' reported age at menarche and age at menopause. Menopausal status was defined based on whether women reported still menstruating (premenopausal) or had stopped menstruating (postmenopausal) at the time of questionnaire completion. Menopausal status and age at menopause were combined into one variable for analyses (premenopausal, <45, 45–49, ≥50 years).

### Covariate Assessment

Detailed information was collected on the type, duration and quantity of tobacco product smoked, including cigarettes with or without filter, bidi, choor or kankat, hooka or pipe, and hashish. Cumulative active smoking (CAS; continuous) was defined as the sum of the total years of use of the above tobacco products. Pack-years of smoking (continuous) was calculated by multiplying years of use by frequency of use (per day) divided by 20 for each tobacco product.

### Statistical Analysis

Descriptive characteristics of the cases and controls were compared using *t*-tests and Chi-square tests. Odds ratios (ORs) and 95% confidence intervals (CIs) for associations between exposures noted above and lung cancer (outcome) were estimated using multivariable logistic regression. Continuous variable measurements were categorized based on distributions among the controls; missing values were categorized as “unknown.”

Based on prior literature, potential confounders were identified *a priori*, including age, smoking history, body mass index (BMI, kg/m^2^), residential history, ethnicity, marital status, education, family monthly income, first degree family history of cancer, history of medical condition (including tuberculosis, asthma and pneumonia), and alcohol consumption. As smoking is a well-established lung cancer risk factor, CAS was included in all models and the above-mentioned potential confounders were subsequently added to determine if estimated ORs changed by more than 10%. After adjusting for CAS, none of the above-mentioned potential confounders changed the estimated ORs by more than 10%. Thus, the final parsimonious models included adjustment for age and CAS, and are presented herein. Additionally, a final model that simultaneously adjusted for all reproductive and hormonal exposure variables of interest, in addition to adjustment for age and CAS, was performed using logistic regression. Model diagnostics were performed for the final models. One subject with missing cumulative active smoking history was excluded from all analyses.

Given the strong relationship between smoking and lung cancer, we also examined associations stratified by cumulative active smoking years. CAS categories (<15 years and ≥15 years), for the stratified analysis, were based on the mean cumulative active smoking years among the controls.

Tests for trend were performed by modeling each categorical exposure as an ordinal variable using logistic regression. The SAS statistical package, version 9.3, was used for all statistical analyses. All *p*-values were 2-sided.

## Results

Descriptive characteristics of the study population are presented by case status (*N* = 267 cases, 226 controls) in Table 1A. Compared to controls, cases were slightly older (mean age (years) ± standard deviation (SD): 59.2 ± 11.6 vs. 53.2 ± 9.6, *p* < 0.0001), less educated (3.8% of cases completed high school vs. 12% of controls, *p* < 0.0001), and had lower family income (25.8% with a monthly family income ≤ 999 rupees compared to 12.8% of controls, *p* = 0.0002). Additionally, cases were more likely to be unmarried or widowed (30 vs. 15.5% of controls, *p* = 0.0002) and to reside in rural areas (86.9% compared to 79.2% of controls, *p* = 0.02). Cases and controls also varied in their ethnic background (*p* < 0.0001); controls had a higher proportion of Brahmins (29.2%) compared to cases (11.6%), and more than fifty percent of cases were included in the “other” ethnic groups.

**Table 1A:** Characteristics of women enrolled in the hospital-based lung cancer case-control study in Bharatpur, Nepal, 2009–2012 (*n* = 493).

	**Cases (*****n*** **=** **267)**		**Controls (*****n*** **=** **226)**		***p*-value^a^**
	**Mean** **±** **SD**				
**Age (years)**	59.2 ± 11.6		53.2 ± 9.6		<0.0001
**BMI (kg/m**^**2**^**)**	20.1 ± 3.5		22.6 ± 4.0		<0.0001
**Years of tobacco product use**					
Cigarettes with filter	5.5 ± 12.5		3.8 ± 10.2		0.1
Cigarettes without filter	24.9 ± 22.5		7.0 ± 13.6		<0.0001
Bidi	12.3 ± 19.5		2.7 ± 8.9		<0.0001
Choor/kankat	4.6 ± 13.0		0.9 ± 5.3		<0.0001
Hooka/pipe & hashish	2.2 ± 10.8		0.5 ± 3.9		0.02
**Cumulative number of active smoking years**	49.6 ± 32.5		15.1 ± 23.2		<0.0001
**Pack-years of smoking**	24.7 ± 28.2		5.5 ± 11.9		<0.0001
	**N**	**%^b^**	**N**	**%^b^**	***p*****-value^c^**
**Ethnicity**					<0.0001
Brahmin	31	11.6	66	29.2	
Chettri	56	21	37	16.4	
Madishe/tharu	29	10.9	22	9.7	
Other	151	56.6	101	44.7	
**Marital status**					0.0002
Married	187	70.0	191	84.5	
Unmarried/widowed	80	30.0	35	15.5	
**Residential area**					0.02
Urban	35	13.1	47	20.8	
Rural	232	86.9	179	79.2	
**Education**					<0.0001
None	242	90.6	157	69.5	
Elementary	15	5.6	42	18.6	
High school	10	3.8	27	12.0	
**Family monthly income (rupees)**					0.0002
≤ 999	69	25.8	29	12.8	
1000–1999	74	27.7	55	24.3	
≥ 2000	124	46.4	142	62.8	
**First degree family history of cancer (yes)**	79	29.6	57	25.2	0.28
**History of tuberculosis (yes)**	30	11.2	11	4.9	0.01
**History of asthma (yes)**	23	8.6	14	6.2	0.3
**History of pneumonia (yes)^d^**	13	4.9	3	1.3	0.04
**Alcohol consumption (ever)**	94	35.2	42	18.6	<0.0001
**Tobacco use (ever)**	234	87.6	97	42.9	<0.0001

*BMI, body mass index; SD, standard deviation; N, number of participants*.

a *P-values generated by t-test*.

b *Percentages may not add up to 100 because of rounding*.

c *P-values generated by chi-square test*.

d *There was one missing value, which was not included in the test*.

In terms of lifestyle factors, cases were significantly more likely to use tobacco products (*p* < 0.0001), consume alcohol (*p* < 0.0001), and to have a slightly lower BMI (*p* < 0.0001). When smoking behaviors were analyzed in more detail, results indicated that cases reported greater cumulative active smoking years (mean ± SD: 49.6 ± 32.5 vs. 15.1 ± 23.2, *p* < 0.0001) and pack-years of smoking (mean ± SD: 24.7 ± 28.2 versus 5.5 ± 11.9, *p* < 0.0001). The difference between cases and controls was not statistically significant for years of filtered cigarette use (*p* = 0.1). Nevertheless, these groups were significantly different with respect to years of use of unfiltered cigarettes (*p* < 0.0001), bidi (*p* < 0.0001), choor or kankat (*p* < 0.0001), and hooka or hashish (*p* = 0.02). Cases were also more likely to report a prior history of tuberculosis (*p* = 0.01) and pneumonia diagnosis (*p* = 0.04).

Distributions of reproductive and hormonal characteristics were examined among cases and controls (Table 1B), with a similar mean age at menarche (*p* = 0.17), and age at first live-birth (*p* = 0.9) observed between both groups. However, a greater proportion of controls reported they were still menstruating (*p* < 0.0001), using birth control (*p* = 0.04), and on average, had fewer pregnancies (*p* = 0.005).

**Table 1B:** Reproductive and hormonal characteristics of women by case status.

	**Cases (*****n*** **=** **267)**		**Controls (*****n*** **=** **226)**		***p*-value^a^**
	**Mean** **±** **SD**				
**Menstrual duration ^b^** **(years)**	31.2 ± 5.2		32.2 ± 5.1		0.09
**Age at menopause ^b^** **(years)**	46.3 ± 5.0		46.9 ± 4.8		0.3
**Age at menarche (years)**	14.9 ± 1.7		14.7 ± 1.6		0.3
**Number of pregnancies (gravidity)**	5.1 ± 2.6		4.4 ± 2.4		0.003
**Age at first birth ^c^** **(years)**	20.0 ± 3.5		19.8 ± 3.3		0.42
**Age at first live birth ^c^** **(years)**	20.1 ± 3.6		19.8 ± 3.4		0.38
	**N**	**%^d^**	**N**	**%^d^**	***p*****-value^a^**
**Still menstruating (yes)**	25	9.4	71	31.4	<0.0001
**Age at menarche (years)**					0.17
<14	46	17.2	50	22.1	
14	58	21.7	62	27.4	
≥15	134	50.2	101	44.7	
**Age at menopause (years)**					<0.0001
Premenopausal	25	9.4	71	31.4	
<45	61	22.9	39	17.3	
45–49	98	36.7	55	24.3	
≥50	71	26.6	52	23.0	
**Number of pregnancies (gravidity)**					0.005
≤ 2	40	15.0	43	19.0	
3–5	116	43.5	121	53.5	
>5	111	41.6	62	27.4	
**Age at first birth (years)**					0.8
Never Pregnant	15	5.6	10	4.4	
<18	50	18.7	47	20.8	
18–20	100	37.5	80	35.4	
≥21	77	28.8	69	30.5	
**Age at first live-birth (years)**					0.9
Never Pregnant	15	5.6	10	4.4	
<18	50	18.7	47	20.8	
18–20	98	36.7	80	35.4	
≥21	79	29.6	69	30.5	
**Birth control use (ever)**	29	10.9	39	17.3	0.04

*SD, standard deviation; N, number of participants*.

a *P-values generated by t-test or chi-square test; unknown category or missings excluded*.

b *Only among postmenopausal women*.

c *Only among women who reported being pregnant*.

d *Percentages may not add up to 100 because of rounding and due to unknown categories (not shown)*.

Among postmenopausal women, those with a younger age at menopause (<45 years and 45–49 years) had an increased odds of lung cancer compared to women who were ≥50 years at menopause [OR (95% CI): 2.14 (1.09, 4.17) and 1.93 (1.07, 3.51), respectively], after adjusting for age and CAS years (Table 2). The linear trend for age at menopause, with respect to lung cancer, was also statistically significant (p-trend: 0.02) suggesting a dose-response relationship. Furthermore, among postmenopausal women, women with a longer menstrual duration had a slightly decreased odds of lung cancer compared to those with a shorter menstrual duration [age and CAS adjusted OR (95% CI): 0.93 (0.88, 0.98)]. Lastly, women who were still menstruating had a decreased odds of lung cancer compared to those who had stopped menstruating [OR (95% CI): 0.41 (0.20, 0.85)]. All other reproductive and hormonal factors examined were not associated with lung cancer, after adjusting for age and CAS.

**Table 2:** Multivariable odds ratios and 95% confidence intervals for the relationship between reproductive and hormonal factors and lung cancer in the hospital-based lung cancer case-control study, 2009–2012.

	**Cases**	**Controls**	**Age & CAS adjusted^a^**
	**N**	**N**	**OR (95% CI)**
**Menstrual duration^b^ (years)**	213	142	0.93 (0.88, 0.98)
**Still menstruating**			
Yes	25	71	0.41 (0.20, 0.85)
No	196	114	1 (referent)
**Age at menarche (years)**			
<14	46	50	0.93 (0.53, 1.62)
14	58	62	0.88 (0.52,1.49)
≥15	134	101	1 (referent)
p-trend^a^			0.77
**Age at menopause ^b^(years)**			
<45	61	39	2.14 (1.09, 4.17)
45–49	98	55	1.93 (1.07, 3.51)
≥50	71	52	1 (referent)
p-trend^a^			0.02
**Number of pregnancies (gravidity)**			
≤ 2	40	43	0.87 (0.48, 1.57)
3–5	116	121	1 (referent)
>5	111	62	1.27 (0.78, 2.08)
p-trend^a^			0.21
**Age at first live-birth (years)**			
Never pregnant	15	10	1.07 (0.38, 3.00)
<18	50	47	0.80 (0.44, 1.44)
18–20	98	80	1 (referent)
≥21	79	69	0.76 (0.45, 1.28)
p-trend^a^			0.76
**Birth control use**			
Ever	29	39	0.79 (0.42, 1.50)
Never	227	177	1 (referent)

*CAS, Cumulative Active Smoking; N, number of participants; OR, odds ratio; CI, confidence interval*.

a *Estimates were adjusted for age and cumulative active smoking years; one subject with missing cumulative active smoking history was excluded*.

b *Premenopausal women not included*.

*Unknown categories were retained in all models but not presented here*.

While the magnitude of the odds of lung cancer among postmenopausal women with a younger age at menopause (<45 years and 45–49 years) remained elevated [OR (95% CI): 1.7 (0.90, 3.4) and OR (95% CI): 1.7 (0.90, 3.2), respectively], the estimates were no longer statistically significant after simultaneous adjustment for all reproductive and hormonal factors (Table 3). No other significant relationships with lung cancer were observed at α = 0.05.

**Table 3:** Mutually adjusted odds ratios and 95% confidence intervals of reproductive and hormonal factors in relation to lung cancer in the hospital-based lung cancer case-control study.

	**Cases**	**Controls**	**Mutually adjusted ^a^**
	**N**	**N**	**OR (95% CI)**
**Age at menarche (years)**			
<14	46	50	1.04 (0.57, 1.87)
14	58	62	0.84 (0.48, 1.46)
≥15	134	101	1 (referent)
**Age at menopause (years)**			
Premenopausal	25	71	0.71 (0.29, 1.70)
<45	61	39	1.74 (0.90, 3.38)
45–49	98	55	1.72 (0.94, 3.15)
≥50	71	52	1 (referent)
**Number of pregnancies (gravidity)**			
≤ 2	40	43	0.82 (0.40, 1.68)
3–5	116	121	1 (referent)
>5	111	62	1.15 (0.69, 1.91)
**Age at first live-birth (years)**			
Never Pregnant	15	10	2.39 (0.55, 10.36)
<18	50	47	0.75 (0.40, 1.41)
18–20	98	80	1 (referent)
≥21	79	69	0.77 (0.45, 1.31)
**Birth control use**			
Ever	29	39	0.82 (0.42, 1.59)
Never	227	177	1 (referent)

*OR, odds ratio; CI, confidence interval*.

a *Estimates were simultaneously adjusted for all reproductive and hormonal exposures listed above, in addition to adjustment for age and cumulative active smoking years; one subject with missing cumulative active smoking history was excluded*.

*Unknown categories were retained in the model but not presented here*.

After stratifying by cumulative active smoking (CAS) years (Table 4), younger age at menopause (specifically <45 years) remained significantly associated with a higher odds of lung cancer among postmenopausal women with a cumulative active smoking history of at least 15 years [OR (95% CI): 2.27 (1.02, 5.04)]. Similarly, among postmenopausal women, the inverse relationship between menstrual duration and lung cancer only remained significant among those who reported smoking for ≥15 cumulative years over their lifetime. Women who reported to be still menstruating had decreased odds of lung cancer within both strata (<15 and ≥15 years) of CAS. Lastly, other factors examined (i.e., age at menarche, number of pregnancies, age at first birth, age at first live-birth and birth control use) were not associated with lung cancer within both strata of cumulative active smoking, similar to the overall analysis.

**Table 4:** Multivariable odds ratios and 95% confidence intervals of the relationship between reproductive and hormonal and lung cancer by smoking status.

	**<15 Cumulative active smoking years**			**≥15 Cumulative active smoking years**		
	**Cases**	**Controls**	**OR (95% CI)^a^**	**Cases**	**Controls**	**OR (95% CI)^a^**
	**n**	**n**		**n**	**n**	
**Menstrual duration ^b^** **(years)**	18	81	0.95 (0.84, 1.07)	194	61	0.92 (0.87, 0.98)
**Still menstruating**						
Yes	17	60	0.30 (0.10, 0.92)	8	11	0.29 (0.09, 0.88)
No	17	61	1 (referent)	179	53	1 (referent)
**Age at menarche (years)**						
<14	11	32	1.68 (0.65, 4.30)	35	18	0.71 (0.35, 1.44)
14	10	45	1.01 (0.39, 2.61)	48	17	0.89 (0.44, 1.78)
≥15	13	64	1 (referent)	120	37	1 (referent)
p-trend^a^			0.32			0.37
**Age at menopause^b^** **(years)**						
<45	6	25	2.46 (0.49, 12.24)	55	14	2.27 (1.02, 5.04)
45–49	10	33	3.33 (0.76, 14.60)	88	22	1.81 (0.92, 3.57)
≥50	3	25	1 (referent)	67	27	1 (referent)
p-trend^a^			0.45			0.03
**Number of pregnancies (gravidity)**						
≤ 2	9	27	1.20 (0.46, 3.13)	31	16	0.62 (0.29, 1.32)
3–5	19	85	1 (referent)	96	36	1 (referent)
>5	10	34	1.88 (0.72, 4.92)	101	28	1.10 (0.60, 2.00)
p-trend^a^			0.48			0.19
**Age at first live-birth (years)**						
Never pregnant	4	5	3.78 (0.81, 17.62)	11	5	0.45 (0.13, 1.51)
<18	9	27	1.90 (0.67, 5.37)	41	20	0.52(0.25, 1.10)
18–20	11	58	1 (referent)	87	22	1 (referent)
≥21	9	42	1.06 (0.39, 2.88)	70	27	0.58 (0.30, 1.14)
p-trend^a^			0.35			0.99
**Birth control use**						
Ever	9	28	1.07 (0.43, 2.65)	19	11	0.63 (0.26, 1.49)
Never	27	115	1 (referent)	200	62	1 (referent)

*OR, odds ratio; CI, confidence interval*.

a *Estimates were adjusted for age and cumulative active smoking years, and exposures listed in the above table as applicable; one subject with missing cumulative active smoking history was excluded*.

b *Premenopausal women not included*.

## Discussion

Findings from this first analysis of reproductive and hormonal factors in relation to lung cancer among Nepali women suggest a potential role of menstrual factors in lung cancer. Specifically, later age at menopause was significantly associated with a reduced odds of lung cancer, after adjusting for age and cumulative active smoking. These findings add to the growing body of literature that point to a complex pattern of association between reproductive hormones and lung cancer and contribute new information regarding these exposures among Nepali women, an understudied population.

Our findings with regard to age at menopause is consistent with the few large prospective cohort studies conducted among mainly Caucasian populations in the United States (US) (20, 21, 23), Canada (16) and Italy (22) that observed an inverse association between lung cancer and later age at menopause. However, mixed results have been observed among studies conducted among Chinese populations. More specifically, two cohort studies among non-smoking women (24, 26) and a case-control study (15), found an inverse association, whereas, a cohort study among female textile workers in Shanghai (25) found no association between age at menopause and lung cancer risk. Different smoking patterns and other life-style factors may play a role in the inconsistent results observed among these populations. Nepali women have been reported to have an average age at menopause between 46.8 and 49.9 years (32–36), which is similar to the average age at menopause observed in the present analysis (cases: 46.3 vs. controls: 46.9 years). The median age at menopause among white women in industrialized countries is reported to be between 50 and 52 years (37). Additional studies may be helpful in clarifying the relationships between these factors and lung cancer among the Nepali women population, with its unique distribution of sociodemographic, lifestyle, smoking and health-related behaviors.

Menstrual duration reflects a woman's reproductive period during which she is exposed to hormones produced and secreted by her ovaries, such as estrogen (38). We observed an inverse association between menstrual duration and lung cancer which is in-line with findings from previous studies that have also examined this relationship (22, 24). More specifically, a cohort study among lifetime non-smokers in China (24) and a case-control study among Italian women (22) also observed a reduced risk of lung cancer among women with a longer reproductive period. Potential mechanisms for this association are unclear, however, reproductive hormones, estrogen and progesterone, are suggested in the development of lung cancer, in part, due to sex differences in the observed incidence of lung cancer by subtype (13, 39). Additionally, evidence supports the presence of receptors for Estrogen-β and progesterone in lung cancer cells (14, 40). Prior studies have also reported that women may be more susceptible to the carcinogenic effects of tobacco (8–10), suggesting hormones may play a role. However, the specific mechanisms whereby duration of endogenous estrogen exposure, as measured by duration of menstruation and age at menopause in our study, influences lung cancer requires further mechanistic studies. Complex combinations of hormonal and environmental exposures, such as tobacco consumption, are likely to impact development of distinct histological subtypes of lung cancer.

Interestingly, a case-control analysis conducted by Koushik et al. among 422 women with lung cancer and 577 controls in Canada found an increased risk of lung cancer among women who had a non-natural menopause (predominantly including women who had a bilateral oophorectomy) compared to women who had a natural menopause (16). Koushik et al. also observed an inverse association between age at menopause and lung cancer risk (16). We were unable to conduct stratified analysis by type of menopause or lung cancer subtypes, or conduct a restricted analysis among non-smoking women only, which could provide further insights to our finding among the Nepali population.

Prior studies that examined age at menarche and lung cancer risk have been inconsistent. We found a null relationship between age at menarche and lung cancer which is consistent with various case-control (16–19) and prospective cohort studies (20, 24–27) conducted among Chinese (18, 24–26), Canadian (16, 27), American (19, 20), and German (17) women populations. However, other studies have also reported an inverse association between age at menarche and lung cancer among Chinese (15) and American (21) women. Based on prior studies, the mean age at menarche among Nepali girls ranges from 12 to 14.8 years of age (41–43). However, despite the older age at menarche among Nepali women in our study (mean ± SD: 14.7 ± 1.6) vs. US women (20) (mean ± SD: 12.5 ± 1.4), we also observed a null association. Our observations among Nepali women add important and much needed information to the existing body of literature in this area.

With regard to gravidity, age at first birth, and birth control use in relation to lung cancer, our null findings are also consistent with the majority of the epidemiologic studies that have examined these associations. Only one prior study has examined the relationship between gravidity and lung cancer (16). Similar to our findings, this population-based case-control study observed no statistically significant association between gravidity and lung cancer (16). Results from the current analysis of age at live-birth and age at first birth are similar to several studies that observed a null relationship between age at first birth and lung cancer (15, 16, 18–20, 22, 24, 25, 28). Conversely, Brinton et al. (21) and Kabat et al. (27) reported an inverse association between age at first birth and lung cancer in the NIH-AARP Diet and Health Study and the Canadian National Breast Screening Study cohorts. Differences in the study design as well as the prevalence of exposures may, in part, explain disparate results. The median age at first birth among Nepali women aged 25–49 years was reported to be 20.2 years based on national data (44), which is similar to the mean age at first birth within our study population (cases: 20.0 ± 3.5 years vs. controls: 19.8 ± 3.3 years). Based on the National Vital Statistics Reports (NVSR), the mean age at first birth for women in the US was 26.8 years in 2017 (45). As no prior study has examined the relationship between age at first birth and lung cancer among Nepali women, our results contribute to the understanding of reproductive risk factors in this unique population with a different exposure pattern. Lastly, the null association observed between birth control use and lung cancer risk in the current study, is consistent with the majority of prior studies (19–22, 24, 27, 29). Although the patterns of gravidity, age at first birth and birth control use among our Nepali population differ from those in the predominantly Caucasian or Chinese populations of the previous studies, the consistency of findings reported across multiple populations lend support to these associations.

The majority of Nepali women, particularly the lung cancer cases, in our analytic population reported smoking (only 12.4% of the cases and 57.1% of the controls never smoked), limiting our ability to assess relationships between reproductive and hormonal exposures and lung cancer among non-smokers. Although we stratified our analysis by cumulative active smoking (<15, ≥15 years), few cases within our analytic population reported smoking <15 CAS years. A recent cross-sectional study among Nepali women 15–49 years of age reported the prevalence of tobacco consumption to be 43.6% (46). However, in the United States (and countries similar), the prevalence of smoking is much lower; 13.5% as reported by the Centers for Disease Control and Prevention (47). Given the long-standing relationship between tobacco smoking and lung cancer, it is not surprising that in our population, lung cancer cases smoked tobacco significantly longer compared to controls (mean (year): 49.6 vs. 15.1; *p* < 0.0001). Furthermore, our data reflect the high prevalence of smoking in this population. Nonetheless, due to the limited sample size within the CAS strata, findings from the smoking stratified analyses should be interpreted with caution and corroborated in a larger sample of non-smoking Nepali women. Additionally, the prevalence of EGFR mutations, ALK rearrangement and ROS1 within our study population is unknown as we have not measured these genetic markers. Associations also could not be analyzed by strata of menopausal status, due to the limited number of premenopausal women in our study population. Data on the type of menopause (natural, surgical, or other) was also not available. This information may help in elucidating relationships between age at menopause and lung cancer. Finally, information on lung cancer histology and specific types of birth control use may have provided additional clarification.

Despite these limitations, this is one of the few epidemiological studies examining potential lung cancer risk factors in Nepal (30, 31, 48–50), a country with limited cancer research resources and is the first study to examine the relationship between reproductive and hormonal factors and lung cancer among Nepali women. Additionally, studies that have examined the prevalence of reproductive and hormonal factors among Nepali women are scarce, and this study contributes much needed prevalence data on these exposures among Nepali women. The use of trained nurses via in-person interviews, standardized questionnaires, and detailed information on type, duration and quantity of tobacco products used are strengths of this study. In addition to contributing to the epidemiological body of evidence surrounding potential associations between reproductive and hormonal factors and lung cancer, this study contributes much needed information on the distribution of these risk factors among Nepali women.

Lung cancer is one of the three most common cancers diagnosed among Nepali women, and thus, it is crucial to understand and characterize the prevalence of risk factors in this population. However, research within the Nepali women population is lacking. Findings from this analysis contribute to our understanding of lung cancer occurring among Nepali women with a different prevalence of reproductive and hormonal factors and a high rate of lung cancer incidence. Further research is need to disentangle the relationship between smoking, reproductive and hormonal factors and lung cancer within this population and to assess the clinical applications of these associations.

## Author Contributions

AS and MH designed and implemented the primary study. CD and SV conceptualized the ancillary study concept. AS, MH, CP, BT, and BS carried out the acquisition and quality control of data. SV performed statistical analysis of data. SV, CD and M-LL interpreted the results. SV wrote and drafted the manuscript. SV, CD, MH, and PW critically revised the manuscript for important intellectual content. All authors agreed to be accountable for the content of the work.

### Conflict of Interest Statement

The authors declare that the research was conducted in the absence of any commercial or financial relationships that could be construed as a potential conflict of interest.
